# AICAR suppresses TNF-α-induced complement factor B in RPE cells

**DOI:** 10.1038/s41598-017-17744-w

**Published:** 2017-12-15

**Authors:** Eun Jee Chung, Nikolaos E. Efstathiou, Eleni K. Konstantinou, Daniel E. Maidana, Joan W. Miller, Lucy H. Young, Demetrios G. Vavvas

**Affiliations:** 1 Retina Service, Angiogenesis Laboratory, Massachusetts Eye and Ear Infirmary, Department of Ophthalmology, Harvard Medical School, Boston, Massachusetts 02114 United States; 2 Department of Ophthalmology, National Health Insurance Service Ilsan Hospital, Gyeonggi-do, Korea

## Abstract

Age related macular degeneration is the leading cause of blindness in the developed world. Although its precise cause remains elusive, dysfunction of the retinal pigment epithelium (RPE) and dysregulation of complement have been implicated in its pathogenesis. The goal of this study was to evaluate the role of an AMP-dependent kinase (AMPK) activator, 5-aminoimidazole-4-carboxamide riboside (AICAR), on tumor necrosis factor alpha (TNF-α) induction of complement factor B (CFB) in RPE cells. We found that AICAR inhibited TNF-α-induced CFB expression in ARPE-19 and human primary RPE cells in a dose-dependent fashion. Treatment of cells with dipyridamole, which blocks AICAR cellular uptake abolished these effects. In contrast, the adenosine kinase inhibitor, 5-iodotubericidin, which inhibits the conversion of AICAR to the direct activator of AMPK, ZMP, did not reverse the effects on TNF-α-induced CFB expression, suggesting AMPK-independent effects. Indeed, knockout of AMPK in RPE cells using Clustered Regularly Interspaced Palindromic Repeats (CRISPR)/Cas9 did not abolish the inhibitory effects of AICAR on RPE CFB expression. Collectively, our results suggest that AICAR can suppress TNF-α-induced CFB expression in RPE cells in an AMPK-independent mechanism, and could be used as a therapeutic target in certain complement over-activation scenarios.

## Introduction

Age-related macular degeneration (AMD) is the leading cause of irreversible vision loss in individuals over 55 years^1^. Non-exudative or dry AMD, the most prevalent form, leads to loss of retinal pigment epithelium (RPE) and subsequent photoreceptor degeneration in the macula. Exudative or wet AMD, although more rare, can account for a significant proportion of cases with severe vision loss due to choroidal neovascularization (CNV), leakage of new vessels, acute hemorrhage, and rapid photoreceptor degeneration^2^. Studies on the pathogenesis of AMD indicate that inflammation is a fundamental component of the disease process, and that the alternative pathway (AP) of complement plays a critical role in driving the inflammatory response. Although polymorphisms in the genes coding for complement factor H (CFH) are the major risk factor for dry AMD, complement factor B (CFB), another key component of the AP, has also been shown to be involved in this disease^3–7^. In addition, Bora *et al*. studied a mouse model of laser-induced CNV and reported that the activation of the factor B-dependent alternative pathway, but not the classical or lectin pathways, was required for the development of CNV^8,9^.

The extra-hepatic biosynthesis of complement is an important checkpoint of local inflammatory responses, especially in tissues that are shielded from plasma components by a blood-tissue barrier such as the retina. Several studies have demonstrated that human RPE cells can synthesize C3, C5, CFH, CFB, factor I, and factor H-related protein (FHL)^10,11^. At these local sites of inflammation, inflammatory cytokines, particularly IFN-γ and TNF-α, regulate CFB expression^12–15^. A previous report demonstrated that TNF-α induced CFB gene expression and the κB *cis*-binding site at −433 to −423 bp was required for TNF-α-stimulated CFB promoter induction^16^. It has also been shown that decreased membrane complement regulators in RPE contributed to RPE damage in AMD and local production of the CFB by the RPE is sufficient to promote laser-induced CNV^17,18^. Altogether, these results indicate that the RPE is not only one of the main local source of complement, but also that complement synthesis in RPE is subject to regulation by several inflammatory cytokines^10,11^.

Adenosine monophosphate (AMP)-activated protein kinase (AMPK) is a serine/threonine kinase that regulates energy homeostasis and metabolic stress^19^. In particular, it acts as a sensor of cellular energy status and maintains the balance between ATP production and consumption. In addition, AMPK, which is expressed in all cell types, exists as a heterotrimer consisting of a catalytic α subunit and regulatory β and γ subunits^20^. The catalytic α subunit of AMPK has two major isoforms, α1 and α2^21–23^. 5-Aminoimidazole-4-carboxamide ribonucleotide (AICAR) is a commonly used activator of AMPK. AICAR enters cells through the adenosine transporter and is quickly phosphorylated to AICAR monophosphate (ZMP). A rise in intracellular ZMP results in the activation of AMPK by mimicking AMP^24^. Recent evidence suggests that AMPK has a wider range of functions, including the regulation of cell growth, cell proliferation, autophagy, angiogenesis, and ocular inflammation^14,25–27^. However, the role of AMPK and its activator AICAR in complement regulation has not been studied up to now. Since chronic inflammation and complement dysregulation are known factors associated with AMD development, in the present study we examined the role of AMPK in the regulation of CFB expression induced by a pro-inflammatory cytokine, TNF-α, in RPE cells.

## Results

### AICAR inhibits TNF-α-induced expression of CFB in RPE cells

**Figure 1:**
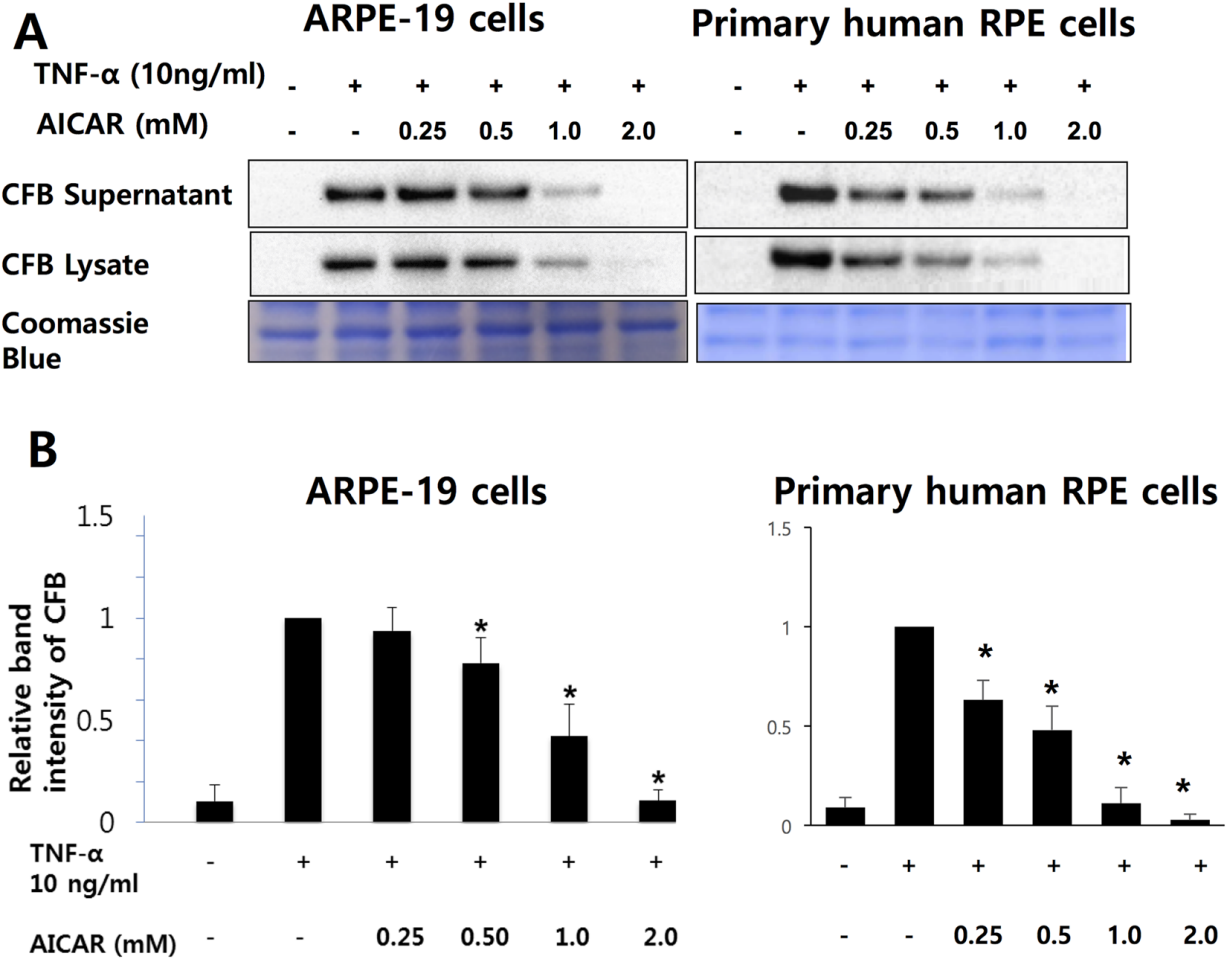
AICAR inhibits TNF-α-induced expression of CFB in RPE cells. (**A**) RPE cells were incubated with different concentrations of AICAR starting 1 hour prior to stimulation with TNF-α (10 ng/mL) for 24 hours. Western blot demonstrating the expression level of CFB in supernatants and cell lysates. Coomassie blue stain indicates the relative loading of the samples. (**B**) Densitometry analysis of CFB expression in RPE cell lysates. Representative blots are shown. *p < 0.05 compared with the TNF-α group.

RPE cell dysfunction has been linked to the development of AMD as a result of the expression of inflammatory mediators and over-activation of complement system. In addition, TNF-α can induce CFB expression in different types of cells including ARPE-19 cells^11,16,28^. After 24 hour-starvation by serum depletion, we treated confluent ARPE-19 and primary human RPE cells with 10 ng/mL of TNF-α for 24 hours, in the presence or absence of various doses of AICAR (0.25–2 mM) and determined CFB expression by Western blot. As seen in Fig. 1, AICAR treatment abrogated TNF-α-induced CFB expression in a dose-dependent manner, in both culture supernatant and cell lysates. Moreover, treatment with 2 mM AICAR halted TNF-α-induced CFB expression to levels similar to baseline, in absence of significant cell death as assessed by MTT assay. (Supplementary Figure 1).

### Dipyridamole but not 5-iodotubercidine abolishes the inhibition of AICAR on CFB expression induced by TNF-α

**Figure 2:**
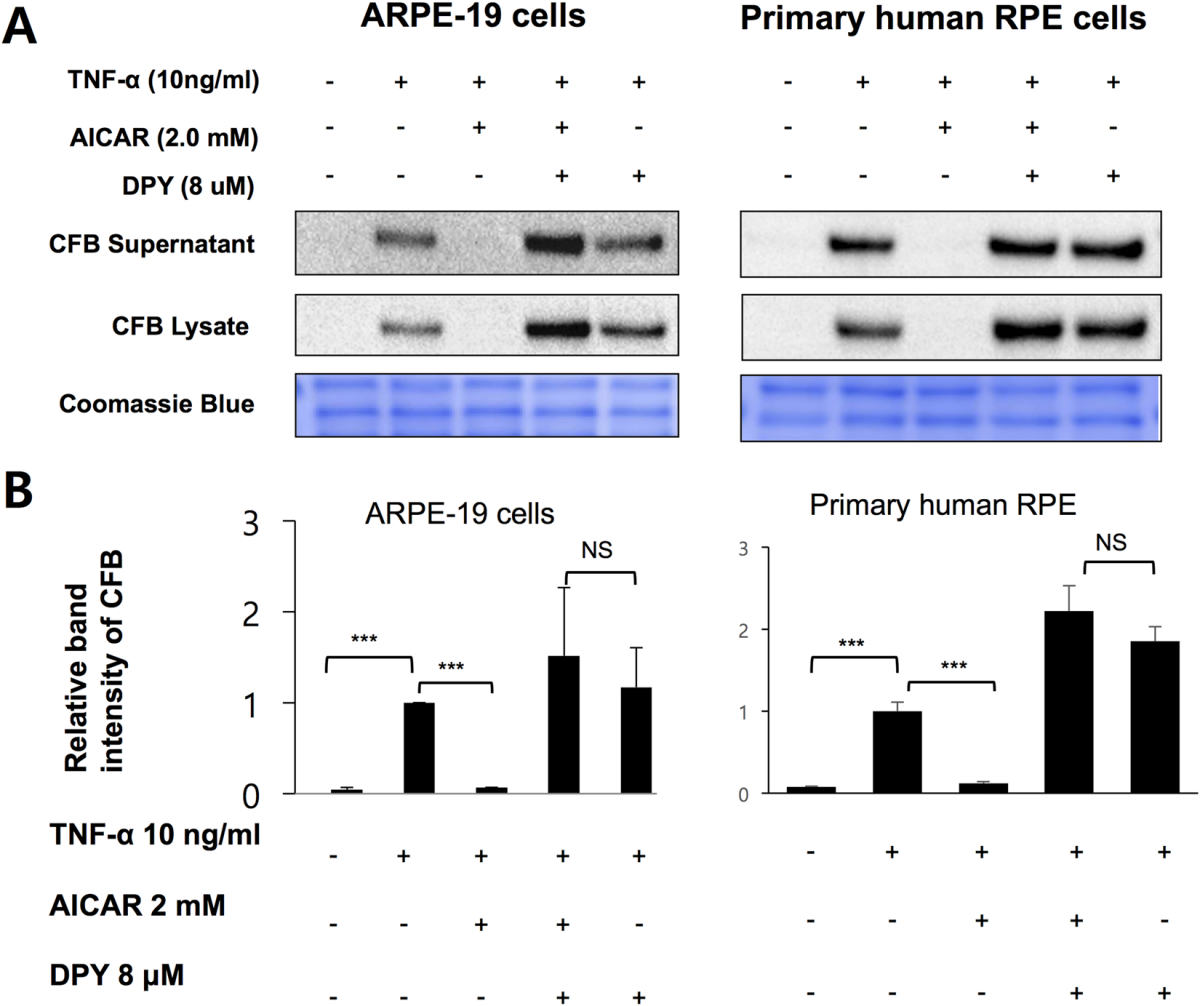
Dipyridamole abolishes the inhibitory effects of AICAR on TNF-α-induced CFB expression. (**A**) RPE cells were preincubated with 8 μM DPY for 1 hour and then treated with 2 mM AICAR for 1 hour followed by 10 ng/mL TNF-α for 24 hours. Western blot demonstrating the expression level of CFB in supernatants and cell lysates. (**B**) Densitometry of CFB in RPE cell lysate in A is shown. Representative blots are shown. *p < 0.05.

**Figure 3:**
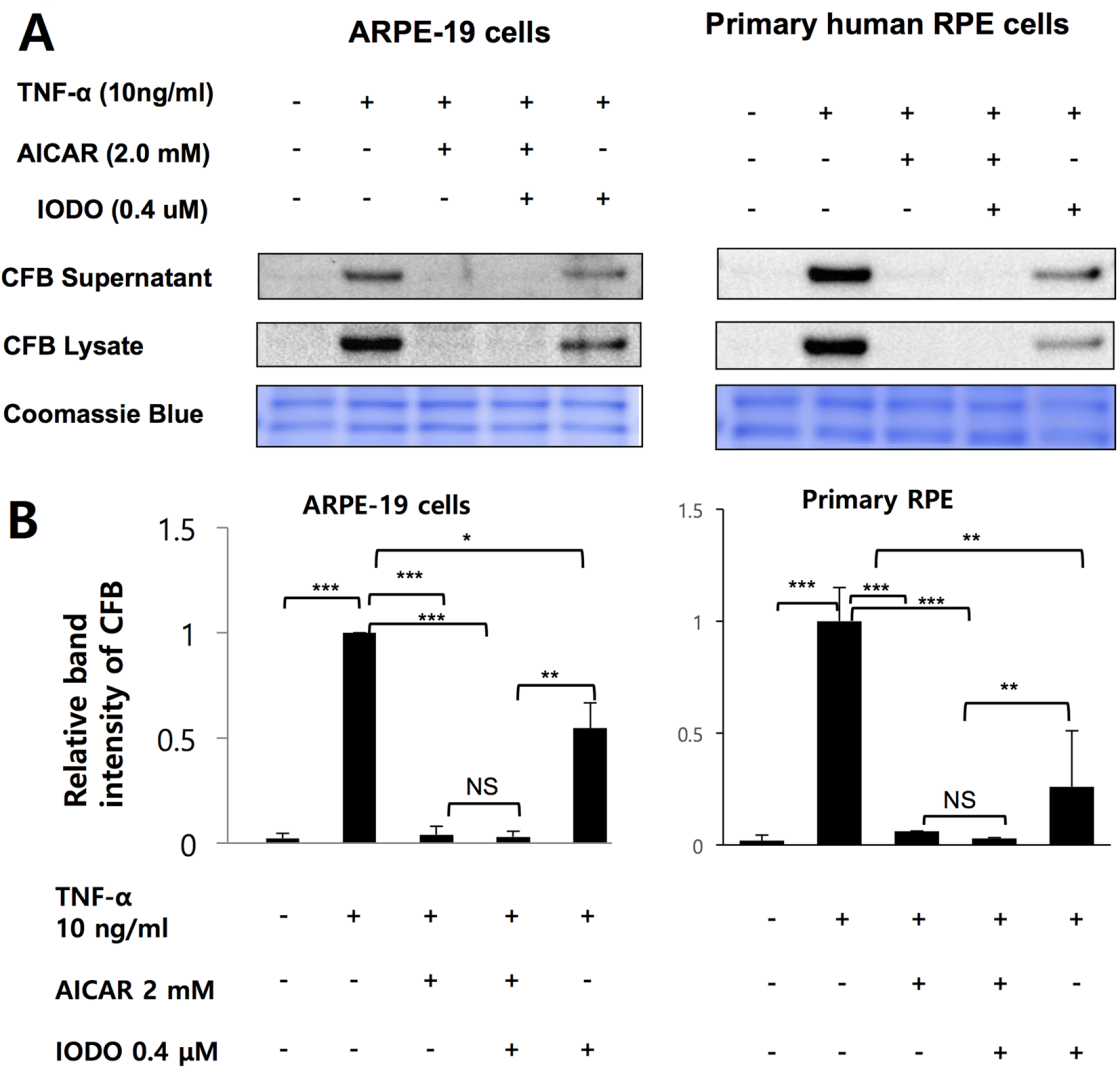
The inhibitory effects of AICAR on TNF-α-induced CFB expression was not affected by 5-IODO. (**A**) RPE cells were preincubated with 0.4 μM IODO for 1 hour and then treated with 2 mM AICAR for 1 hour followed by 10 ng/mL TNF-α for 24 hours. Western blot demonstrating the expression level of CFB in supernatants and cell lysates. (**B**) Densitometry of CFB in RPE cell lysate in A is shown. Representative blots are shown. *p < 0.05.

AICAR, an AMPK activator, is taken up into cells by a nucleoside transporter and phosphorylated by adenosine kinase to its mono-phosphorylated form (ZMP), which consequently activates AMPK^24^. First, we confirmed that AICAR induced AMPK activation in RPE cells in presence of TNF-α, as seen in a Supplementary Figure 2A ^29–36^. To determine whether the inhibitory effect of AICAR on TNF-α-induced CFB expression is mediated by AMPK activation, we used two different small molecule AICAR inhibitors, dipyridamole (DPY) and 5-iodotubercidine (IODO). DPY blocks adenosine transporters and prevents uptake of AICAR into the cells^26,37^. 5-IODO inhibits adenosine kinase in the cell and prevents conversion of AICAR to ZMP, which activates AMPK^26,37^. As expected, blocking the entrance of AICAR into the cells by DPY, or its conversion to the ZMP activator of AMPK by 5-IODO abolished the AICAR effect on AMPK activation (Supplementary Figure 2B and 3). However, only pretreatment with DPY (Fig. 2), and not by 5-IODO (Fig. 3), abolished the effect of AICAR on TNF-α-induced CFB expression. These findings suggest that although AICAR requires cellular uptake to abrogate CFB expression, it does not require conversion to ZMP (the direct analog of AMP and activator of AMPK) to suppress TNF induced CFB expression.

### Inhibition of TNF-α-induced CFB expression by AICAR is independent of AMPK activation

**Figure 4:**
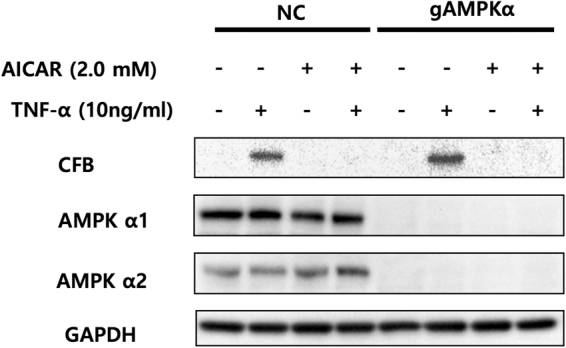
Knockout of AMPKα1 and α2 expression in cultured ARPE-19 cells did not affect the inhibitory effect of AICAR on TNF-α-induced CFB expression. ARPE-19 cells were transfected with guide RNA-Cas9 leading to specific knockout of the AMPKα1 and α2 (gAMPKα), or transfected with a CRISPR/Cas9 vector guided by scramble RNA (NC; negative control). Both groups were incubated with 2.0 mM of AICAR starting 1 hour prior to stimulation with TNF-α (10 ng/mL) for 24 hours. The expression level of CFB and AMPK α1 and α2 were then examined by Western blot. Representative blots are shown.

To acquire further evidence that AICAR effects on CFB expression are independent of AMPK, we knocked out both catalytic isoforms of AMPK (α1 and α2) genes by CRISPR-Cas9 endonuclease system. First we made sure that TNF-α-induced expression of CFB in AMPK α-knockout cells was comparable to that in negative controls using CRISPR/Cas9 vectors guided by scramble RNA (Fig. 4). AMPKα deletion did not modify the inhibitory effect of AICAR on TNF-α-induced CFB expression (Fig. 4). Taken together these results indicate that the catalytic alpha isoform of AMPK is not required for AICAR inhibitory effect on CFB expression in RPE cells.

## Discussion

Prior studies on anti-inflammatory effects of AICAR and AMPK have not examined effects on complement components. The current study shows the ability of AICAR to inhibit TNF-α-induced CFB expression in human RPE cells. Although AICAR is a potent activator of AMPK and many of its effects are mediated by AMPK activation, AMPK-independent effects have also been reported^38–42^. In our study, both pharmacologic inhibition and genetic (CRISPR/Cas9) deletion experiments demonstrate that the effects of AICAR on CFB to be ZMP- and AMPK-independent.

Studies on AMD pathogenesis indicate that inflammation is a fundamental component of the disease process, and that the alternative complement pathway plays a critical role in driving the inflammatory response. Although CFH is the major complement component implicated in AMD, genetic studies have identified variations in the CFB, C2, and C3, complement inhibitory protein factor H, and in the complement activation proteins as major risk factors for AMD^3,4,6,43,44^. Complement component 2 (C2) is paralogous to CFB and resides adjacent to *FB* on chromosome 6p21.3, and haplotypes in *BF* and *C2* have been linked to AMD. L9H *BF*/E318D *C2* and R32Q *BF*/intronic variants of *C2* have been shown to be protective for AMD as leading to impairment in the complement activating function of CFB^4^.

CFB, a factor required to form C3 and C5 convertase, is a serum protein produced mainly by the liver, but not exclusively^4,45,46^. In the eye, CFB can be synthesized by choroid, RPE, and neural retina^4,47^. Interestingly, CFB protein was also detected in ocular drusen and Bruch’s membrane^4^. Previous studies suggested that a complement regulatory framework is present at the retinal/choroidal interface and that the RPE is one of the important regulators of this system^47,48^. In this study, we observed induction of CFB expression by a pro-inflammatory cytokine, TNF-α, in RPE cells (Fig. 1A). This finding is consistent with previous studies showing that inflammatory cytokines such as TNF-α and IFN-γ can affect complement component synthesis^12–15^. TNF-α has also been shown to down-regulate CFH and up-regulate CFB production in RPE cells^47–49^.

Pretreatment of AICAR abolished TNF-α induced CFB expression in a dose-dependent manner although in presence of AMPK activation (Fig. 1). A pharmacologic inhibitor of AICAR entry into cell, DPY, reversed the inhibitory effect of AICAR. In contrast, 5-IODO, an inhibitor of the enzyme responsible for the conversion of AICAR into the direct AMPK activator, ZMP, had negligible effects. Although the DPY and TNF-α co-treatment seemed to increase CFB in RPE cells (Fig. 2), DPY alone did not affect the CFB expression in a further experiment. (Supplementary Figure 4) Interestingly, 5-IODO treatment reduced CFB expression even when treated alone, suggesting intrinsic pharmacologic inhibition of CFB expression in RPE cells (Fig. 3). Furthermore, knockout of AMPKα by CRISPR-Cas9 endonuclease also failed to reverse the inhibitory effect of AICAR on CFB, lending further support that the observed AICAR effects were AMPK independent. Other studies have also shown AICAR to be able to inhibit components of inflammation in an AMPK-independent manner; however, the exact molecular mechanisms still remain unclear^40,50,51^. In one of those studies it was thought that the inhibitory effects of AICAR on cytokine production and ICAM-1 expression were not associated with AMPK but could be attributed to the interference of adenosylmethionine-dependent methylation^40^. In another study, the anti-inflammatory effects of AICAR against LPS-induced iNOS and COX-2 gene transcription were not associated with AMPK activation, but were suggested to result from direct interference of DNA binding to transcriptional factors^51^.

In conclusion, our study shows for the first time the effects of AICAR on complement regulation, abrogating TNF-α-induced CFB expression in RPE cells. This inhibition requires intracellular translocation of AICAR. However, pharmacologic and genetic evidence demonstrated that AICAR inhibitory effects on TNF-α induced CFB are AMPK-independent. Collectively, this suggests that AICAR could be used as a regulator of CFB, yet further experiments are required to elucidate the AMPK independent anti-inflammatory mechanism of AICAR in complement regulation in the RPE.

## Methods

### Materials

RtEGM Retinal Pigment Epithelial Cell Growth Medium (RtEGM BulletKit #195409) was purchased from Lonza (Walkersville, MD) and fetal bovine serum (#10438034) were purchased from Thermo Fisher Scientific (Waltham, MA). CFB antibody (sc-271636) was purchased from Santa Cruz Biotechnology (Santa Cruz, CA). Antibodies for AMPK a1 (Abcam) : 32047,AMPKa2 (Abcam) : 3760, were from Abcam  and (P-ACC (#3661), ACC (#3676), AMPK (#2603), P-AMPK (# 2535), and GAPDH (#2118)  were purchased from Cell Signaling Technologies (Beverly, MA). AICAR (#A611700), a pharmacological activator of AMPK, was purchased from Toronto Research Chemicals (Toronto, ON, Canada). 5-iodotubericidin (IODO #I100), dipyridamole (DPY #D9766) and nicotinamide (N3376) were purchased from Sigma (St. Louis, MO). TNF-α  (#210-TA-020) was purchased from R & D Systems (Minneapolis, MN).

### Cell culture

A human RPE cell line, ARPE-19 and primary human RPE cells were used for the experiments. Cells were cultured and maintained in RtEGM medium supplemented with 1% FBS, 20 mM nicotinamide and 1% penicillin/streptomycin in a humidified incubator with 5% CO_2_ at 37 °C. Experiments were performed on ARPE-19 cells between passages 10 to 20 and human primary RPE cells between passages 2–5, both grown to 90–100% confluence. Upon confluence, serum was depleted for 24 hours and then cells were treated accordingly.

### Protein Extraction and Western Blotting

RPE cells (2.5 × 10^5^/well, 6-well plate) were seeded and cultured for 3 days. Before treatment with TNF-α and/or AICAR, cells were serum-starved for 24 hours in serum-free medium. After treatment, cells were washed with cold PBS and lysed in NP40 cell lysis buffer (Invitrogen, Carlsbad, CA) containing protease and phosphatase inhibitor cocktail (Roche, Indianapolis, IN). Samples were loaded onto a NuPAGE 4–12% Bis-Tris Gel (Novex, Carlsbad, CA), transferred to a polyvinylidene difluoride (PVDF) membrane (0.45 μm; Millipore, Billerica, MA), blocked with 5% non-fat dry milk, and incubated with appropriate primary antibodies. Blots were subsequently incubated with secondary antibodies and images were developed using chemiluminescent substrate (ECL Select western blotting detection reagents, GE Healthcare Life Sciences, Piscataway, NJ). Band signals were detected by an image-scanning densitometer (ChemiDoc imaging system; Bio-Rad) and quantitated by ImageJ 2.0.

### Measurement of cell viability by MTT assay

The effect of various doses of AICAR on cell cytotoxicity was studied using 3-(4,5-dimethylthiazol-2-yl)-2,5-diphenyltetrazolium bromide (MTT). RPE cells were cultured in 96-well plates with 300 μL (1.6*10^4^/mL) of cells in each well and left overnight. Upon confluence, we changed medium with serum free for 24 h. We then pretreated with various concentrations of AICAR (0–2.0 mM) for 1hr and 10 ng/mL of TNF-α for 24 h. The medium was replaced with PBS, containing 0.5 mg/mL MTT (Sigma Aldrich, St. Louis, MO), and cultured at 37 °C for 4 h. 150 μL of dimethyl sulfoxide (DMSO) were mixed with cells and shaken for 10 min. The optical density (OD) at 595 nm was measured using a microplate reader (Molecular Devices, Sunnyvale, CA).

### Silencing of AMPKα expression by CRISPR/Cas9

The knockout of AMPKα1 and/or α2 in ARPE-19 cells was performed by clustered regularly interspaced short palindrome repeats (CRISPR)/Cas9 guided genome editing. Three CRISPR targeting sequences were designed based on the Optimized CRISPR Design web tool (http://crispr.mit.edu), and listed in Table 1. Oligos were cloned into the pSpCas9 (BB)-2A-Puro (PX459) (Addgene, plasmid #62988) following the CRISPR-Cas9 genome engineering protocol by Ran *et al*.^52^. ARPE-19 cells were plated around 75% of confluence 48 hours before transfection in a 12-well plate. Subsequently cells were transfected with three different plasmids (each plasmid contained a single guideRNA). Lipofectamine 3000 Reagent (Invitrogen, L3000008) was used for transfection, as per manufacturer protocol. Cells were treated in media containing 3 μg/mL puromycin (Santa Cruz sc-108071B) for three days. Following selection, culture media was changed every day. From the selected colonies, we further carefully picked one colony for clonal expansion. The AMPKα knockout status of the colony was assessed by Western blot analysis in order to validate sufficient knock-out of the target gene.

**Table 1:** The CRISPR targeting sequences design based on the Optimized CRISPR Design web tool.

AMPKα1	Sequence	Score	Targeted exon
Guide #1	5′-CACCGAAGATCGGCCACTACATTC-3′	90%	1
Guide #2	5′-CACCGATTCGGAGCCTTGATGTGGT-3′	80%	2
Guide #3	5′-CACCGCAGATGGTGTACTGATGACC-3′	74%	3
**AMPKα2**	**Sequence**	**Score**	**Targeted exon**
Guide #1	5′-CACCGAAGATCGGACACTACGTGC-3′	94%	1
Guide #2	5′-CACCGCTGGGCGACACGCTGG GCGT-3′		
	84%	1	
Guide #3	5′- CACCGATTCGCAGTTTAGATGTTGT -3′	76%	2
**AMPKα1 + α2**	**Sequence**	**Score**	**Targeted exon**
Guide #1	5′-CACCGAAGATCGGCCACTACATTC-3′	90%	Exon 1 for AMPKα1 gene
Guide #2	5′-CACCGATTCGGAGCCTTGATGTGGT-3′	80%	Exon 2 for AMPKα1 gene
Guide #3	5′-CACCGAAGATCGGACACTACGTGC-3′	94%	Exon 1 for AMPKα2 gene
Guide #4	5′-CACCGCTGGGCGACACGCTGGGCGT-3′	84%	Exon 1 for AMPKα2 gene

### Statistical analyses

All experiments were repeated a minimum of three times. The data are presented as the mean ± standard deviation (SD). Statistical significance was assessed by Student’s two-sample *t-*tests. Differences were considered significant at *p* < 0.05.

## Electronic supplementary material

Supplementary information
