# Supplementary material for: AICAR suppresses TNF-α-induced complement factor B in RPE cells
Source: Sci Rep. 2017 Dec 15;7:17651. doi: 10.1038/s41598-017-17744-w (PMC5732305; doi:10.1038/s41598-017-17744-w)
Supplement: Supplementary file 1 — Supplementary information [file 41598_2017_17744_MOESM1_ESM.doc]

**AICAR suppresses TNF-α-induced complement factor B in RPE cells**

Eun Jee Chung*^1, 2^, Nikolaos E. Efstathiou*^1^, Eleni K. Konstantinou^1^, Daniel E. Maidana^1^, Joan W. Miller^1^, Lucy H. Young^1^, and Demetrios G. Vavvas^1^

^1^ Retina Service, Angiogenesis Laboratory, Massachusetts Eye and Ear Infirmary, Department of Ophthalmology, Harvard Medical School, Boston, Massachusetts 02114

^2^ Department of Ophthalmology, National Health Insurance Service Ilsan Hospital, Gyeonggi-do, Korea

* Equal contribution

**Correspondence: Demetrios G. Vavvas M.D., Ph.D.**

Tel: 617-573-6874

Fax: 617-573-3011

E-mail: Demetrios_Vavvas@meei.harvard.edu


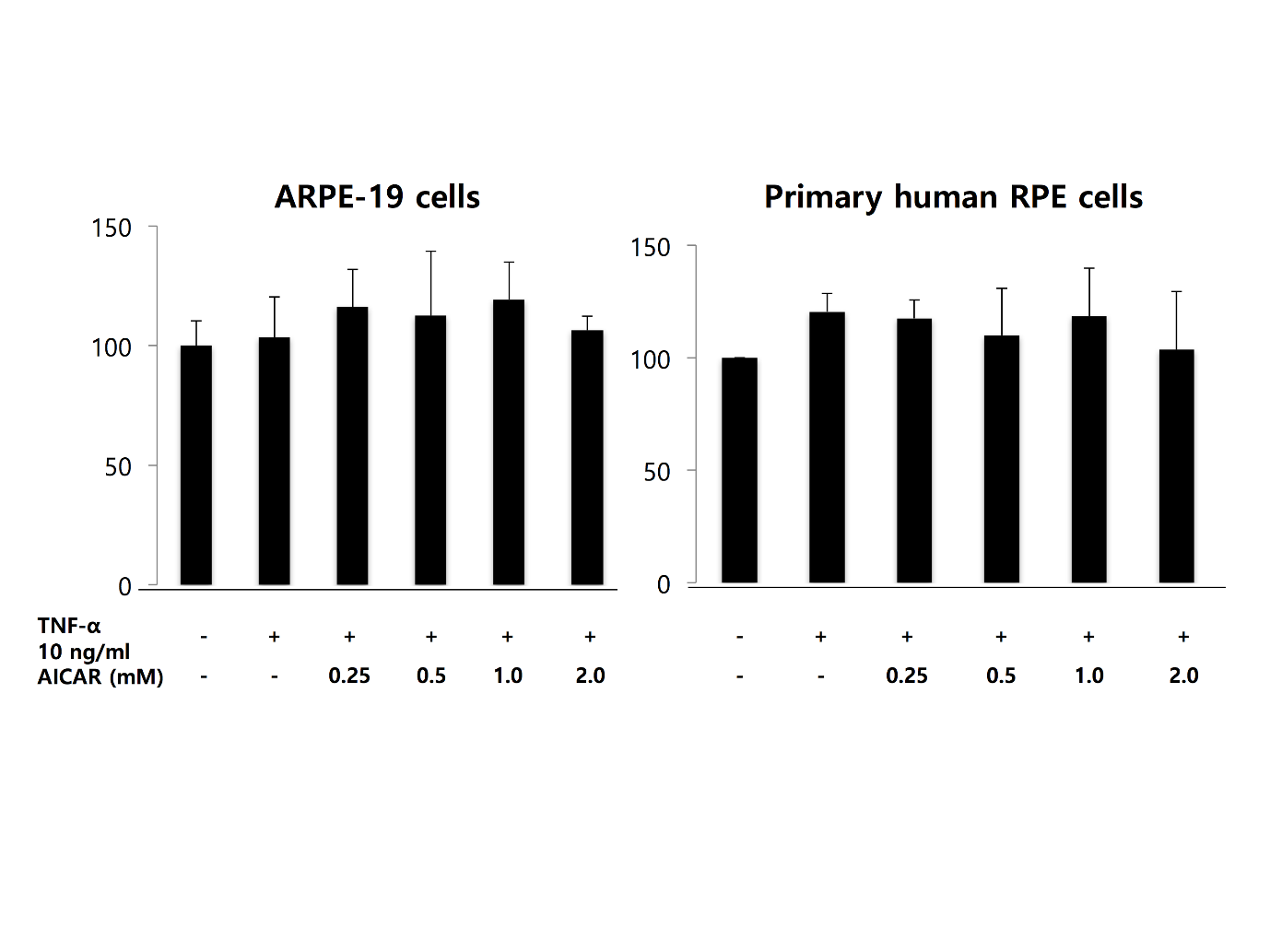
**Supplementary Figure 1** ARPE-19 and human primary RPE cells were incubated with different concentrations of AICAR starting 1 hour prior to stimulation with TNF-α (10 ng/mL) for 24 hours, and cell viability was measured by MTT.


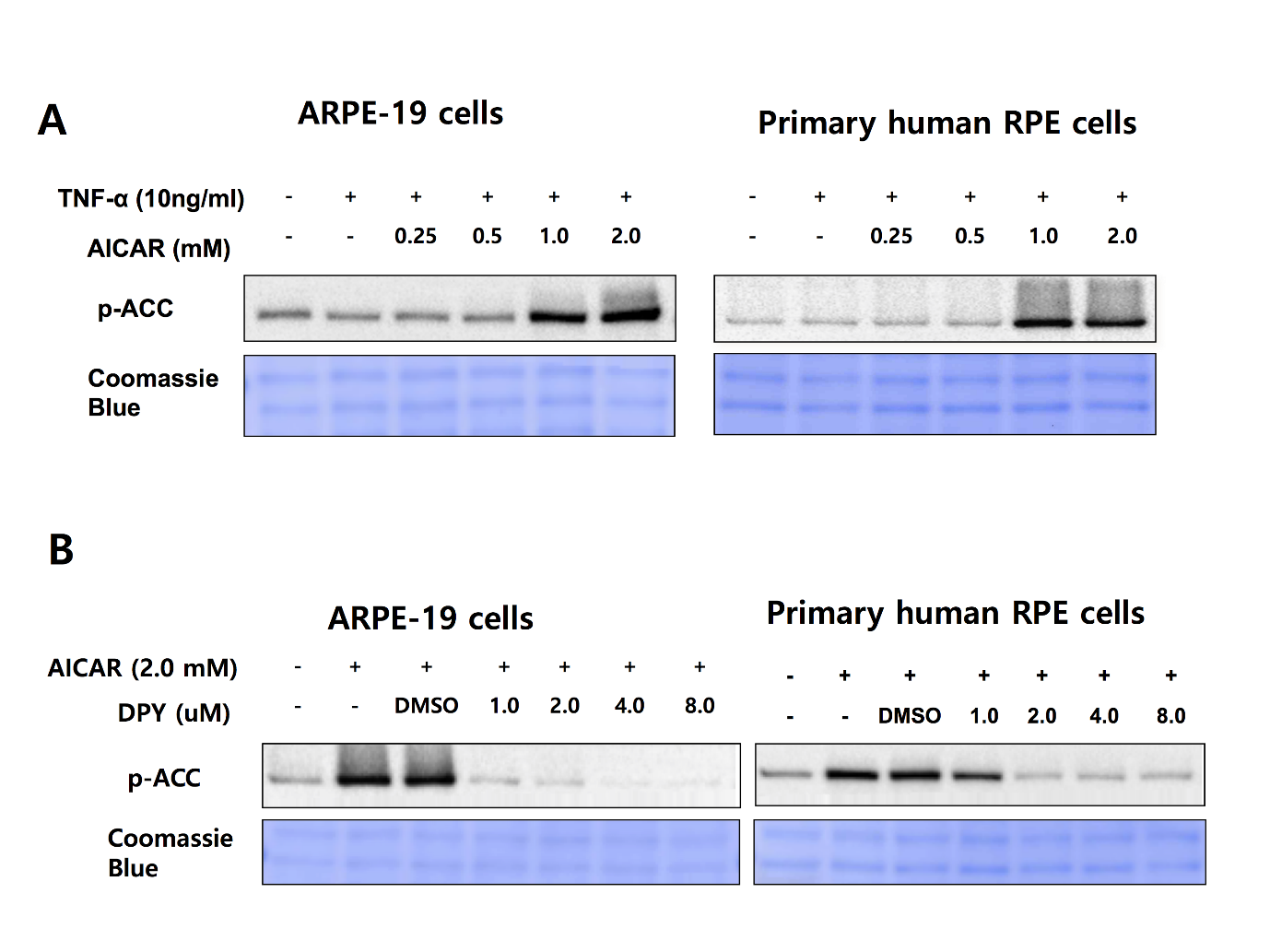


**Supplementary Figure 2** AICAR induces the AMPK acitvation and various doses of DPY abolished its effect. A) RPE cells were treated with various doses (0-2.0mM) of AICAR starting 1 hour prior to stimulation with TNF-α (10 ng/mL) for 24 hours to determine AMPK activation (p-ACC). B) RPE cells were preincubated with several DPY concentrations (1 – 8 µM) or vehicle (DMSO) for 1 hour and then treated with 2.0 mM AICAR for 24 hours to assess AMPK activation (p-ACC).


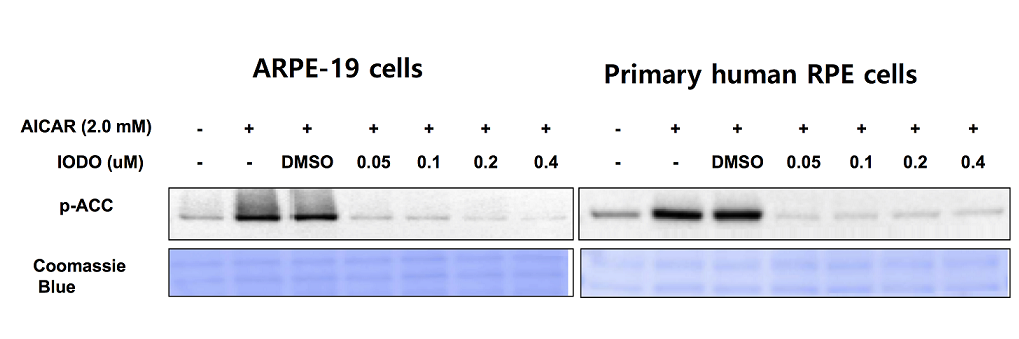


**Supplementary Figure 3** IODO treatment abolished AICAR-induced AMPK activation. A) RPE cells were incubated with several IODO concentrations (0.05 – 0.4 µM) or vehicle (DMSO) starting 1 hour prior to treatment with 2.0 mM AICAR for 24 hours to determine AMPK activation (p-ACC).


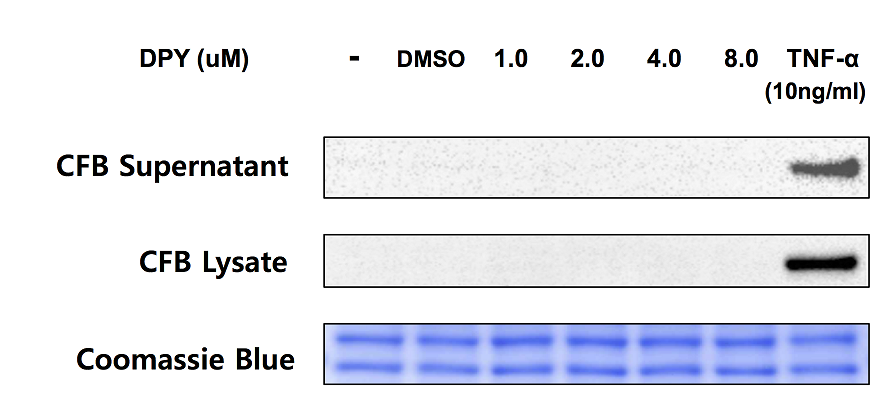


**Supplementary Fig 4** Human primary RPE cells were incubated with different concentrations of DPY or TNF-α (10 ng/mL) for 24 hours. Western blot demonstrating the expression level of CFB in supernatants and cell lysates. Coomassie blue stain indicates the relative loading of the samples.
